# Multidetector CT of iatrogenic and self-inflicted vascular lesions and infections at the groin

**DOI:** 10.1007/s13244-018-0613-6

**Published:** 2018-04-19

**Authors:** Massimo Tonolini, Anna Maria Ierardi, Gianpaolo Carrafiello, Domenico Laganà

**Affiliations:** 1 Department of Radiology, “Luigi Sacco” University Hospital, Via G.B. Grassi 74, 20157 Milan, Italy; 2 Diagnostic and Interventional Radiology Department, ASST Santi Paolo e Carlo, Via A di Rudinì 8, 20142 Milan, Italy; 3 Department of Radiology, “Magna Grecia” University, Viale Europa, 88100 Catanzaro, Italy

**Keywords:** Vascular access, Femoral artery, Complications, Pseudoaneurysm, Computed Tomography (CT)

## Abstract

**Abstract:**

The number and complexity of endovascular procedures performed via either arterial or venous access are steadily increasing. Albeit associated with higher morbidity compared to the radial approach, the traditional common femoral artery remains the preferred access site in a variety of cardiac, aortic, oncologic and peripheral vascular procedures. Both transarterial and venous cannulation (for electrophysiology, intravenous laser ablation and central catheterisation) at the groin may result in potentially severe vascular access site complications (VASC). Furthermore, vascular and soft-tissue groin infections may develop after untreated VASC or secondarily to non-sterile injections for recreational drug use. VASC and groin infections require rapid diagnosis and appropriate treatment to avoid further, potentially devastating harm. Whereas in the past colour Doppler ultrasound was generally used, in recent years cardiologists, vascular surgeons and interventional radiologists increasingly rely on pelvic and femoral CT angiography. Despite drawbacks of ionising radiation and the need for intravenous contrast, multidetector CT rapidly and consistently provides a panoramic, comprehensive visualisation, which is crucial for correct choice between conservative, endovascular and surgical management. This paper aims to provide radiologists with an increased familiarity with iatrogenic and self-inflicted VASC and infections at the groin by presenting examples of haematomas, active bleeding, pseudoaneurysms, arterial occlusion, arterio-venous fistula, endovenous heat-induced thrombosis, septic thrombophlebitis, soft-tissue infections at the groin, and late sequelae of venous injuries.

**Teaching Points:**

• *Complications may develop after femoral arterial or venous access for interventional procedures*.

• *Arterial injuries include bleeding, pseudoaneurysm, occlusion, arteriovenous fistula, dissection*.

• *Endovenous heat-induced thrombosis is a specific form of iatrogenic venous complication*.

• *Iatrogenic infections include groin cellulitis, abscesses and septic thrombophlebitis*.

• *CT angiography reliably triages vascular access site complications and groin infections*.

## Introduction

The number of endovascular procedures performed via either arterial or venous access by cardiologists, vascular surgeons and interventional radiologists has been steadily increasing over the past decades. Percutaneous transarterial access represents the initial step in a variety of cardiac, peripheral vascular, aortic and oncologic procedures. The radial approach is associated with lower morbidity and is therefore recommended by the European Society of Cardiology [1]. However, the traditional common femoral artery (CFA) access remains the preferred technique by many operators due to its easiness and familiarity, particularly when the radial access is unfeasible or large-bore access is needed. Unfortunately, piercing into the arterial system carries the potential risk of vascular access site complications (VASC) that result in prolonged hospitalisation, higher costs, increased morbidity and, occasionally, mortality [2–6].

Alternatively, VASC at the groin may occur following endovascular procedures performed via venous access such as electrophysiology, intravenous laser ablation, or short- or mid-term central venous catheter (CVC) placement. Unfortunately, compared to subclavian veins the femoral venous access suffers from a higher rate of thrombosis and bacterial colonisation [7–10]. Furthermore, vascular and soft-tissue inguinal infections may develop following unrecognised VASC, particularly with use of vascular closure devices (VCD) [11–13] and secondarily to non-sterile injections performed for recreational drug use [14, 15].

Potentially severe vascular and soft-tissue injuries require rapid diagnosis and proper treatment to limit the associated morbidity and avoid further complications. Traditionally, colour Doppler ultrasound (CDUS) was used to investigate suspected VASC, and CT was reserved for those patients with inconclusive sonographic findings. However, in our experience cardiologists and interventional radiologists increasingly rely on pelvic and femoral CT angiography to rapidly and consistently provide a panoramic, comprehensive visualisation of both vascular and infectious complications, which proves crucial for a correct choice between conservative, endovascular and surgical management. This paper aims to provide radiologists with an increased familiarity with iatrogenic and self-inflicted VASC and infections at the groin.

## Complications of femoral arterial access

### Incidence and risk factors

In the cardiologic literature, clinically significant VASC have been reported to occur after 0.8–1.8% of diagnostic coronary angiography (CA) and up to 9% of coronary interventions (CI). Summarised in Table 1, the commonest VASC of CFA access include bleeding, pseudoaneurysm (PA), arterio-venous fistula (AVF), CFA occlusion and dissection. Other occasional complications include local lymphatic leakage and femoral neuropathy secondary to large haematomas or PA [2–5, 16].

**Table 1:** Vascular complications of femoral arterial access (in descending order of frequency)

Complication type	Frequency (% of procedures)
Haemorrhage	4.5% - 12%
Retroperitoneal haemorrhage	0.1% - 0.5%
Femoral pseudoaneurysm	0.05% - 2% - Diagnostic angiography 3.5% - 8% - Interventions
Arterio-venous fistula	<1%
Femoral artery occlusion	0.19%
Femoral artery dissection	0.02–0.3%

In recent years, the increasing use of ultrasound guidance has resulted in significantly decreased (roughly 50%) overall complications and bleeding rates, particularly in elderly and obese patients [6, 17].

General predisposing factors related to VASC development include operator experience, technical and patient-related conditions. Meticulous technique is crucial to minimising risks during transarterial interventions, including correct choice of access site, proper closure, and appropriate management of antithrombotic medications. Repeated puncture attempts and arterial access distal or proximal to the CFA bifurcation are associated with higher risk. The key technical issue is represented by use of large catheters (>6 French) [2, 3].

Patient-related risk factors include female gender, high body mass index, advanced age, low platelet count, hypertension and severity of peripheral atherosclerotic disease. Due to smaller vessels and higher comorbidities, women have a risk of complications double that of men. Left-sided CFA cannulation carries a four times higher risk, probably related to severe atheromatous disease and previous failed right-sided puncture [2–5, 16].

### Haematomas and active bleeding

Haemorrhage represents the commonest form of VASC after transarterial diagnostic and interventional procedures. The degree of severity is quantified by the haemoglobin level drop, and blood transfusions are required in 1% to 6% of patients. Minor bleeding is generally apparent as persistent ecchymosis radiating from the puncture site. Major haemorrhage manifests with a combination of haemodynamic instability, dropping haemoglobin, abdominal pain, neurologic symptoms and swelling of the ipsilateral thigh. Arterial puncture above the inguinal ligament may lead to the uncommon retroperitoneal haemorrhage, which is associated with higher need for transfusions, longer hospital stay and non-negligible mortality (6.6%) [2, 5, 6, 16, 18].

**Fig. 1:**
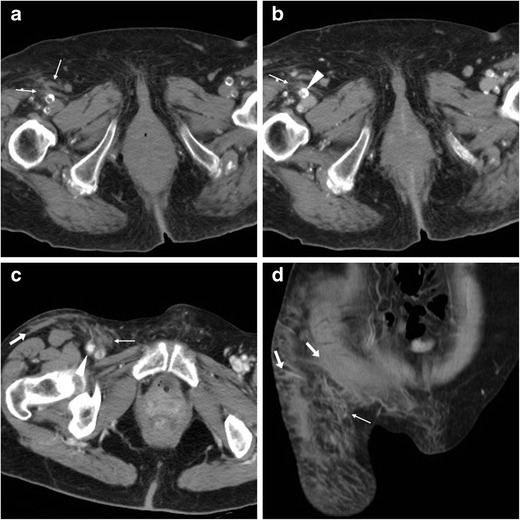
Expected CT appearance following uncomplicated percutaneous access to the right common femoral artery (CFA) in two different patients. In an 82-year-old diabetic female patient, 48 h after coronary angiography (CA) and coronary artery stenting, focused precontrast (A) and arterial-phase (B) CT images showed mild fat stranding and fluid (thin arrows) along the probable puncture tract, abutting the ventral aspect of the calcified but patent CFA (arrowheads). The day after CA, in a 90-year-old female patient, CT-angiography (C, D) showed similar changes (thin arrows) surrounding the CFA (arrowhead) and high-attenuation fascial blood (arrows), which did not require treatment

In our experience, multidetector CT angiography is beneficial in patients with suspected groin VASC, particularly when there is concern for deep-seated haemorrhage [16]. The CT protocol should include a preliminary unenhanced acquisition extending at least from the iliac crests to the mid-thigh, which allows assessing presence, site, and entity of extravascular blood. The expected early CT appearance following uncomplicated transarterial access is represented by fat stranding and fluid abutting the ventral aspect of the punctured CFA, and mild high-attenuation fascial blood (Fig. 1).

**Fig. 2:**
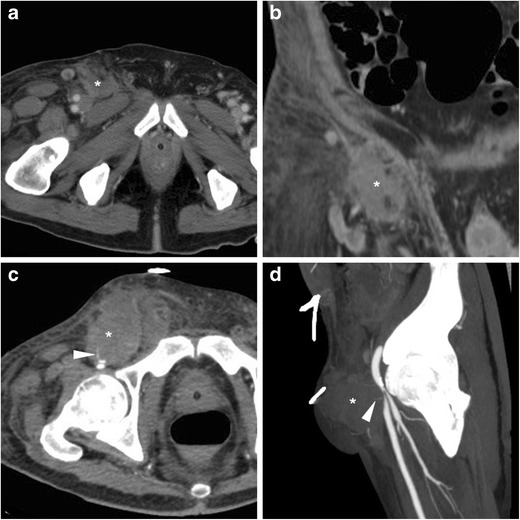
Delayed groin bleeding in a 70-year-old male patient after transarterial mitral and aortic valve replacement. Early CT (A, B) showed circumscribed effusion (*) abutting the CFA at the level of percutaneous access, which was interpreted as a minor bleed and treated conservatively. However, during hospitalisation for infected sternal dehiscence, repeated CT angiography (C, D) showed development of a 4.5-cm groin haematoma (*) containing a thin contrast medium (CM) extravasation focus (arrowheads), causing compression on the CFA (MIP image D) which required stenting

**Fig. 3:**
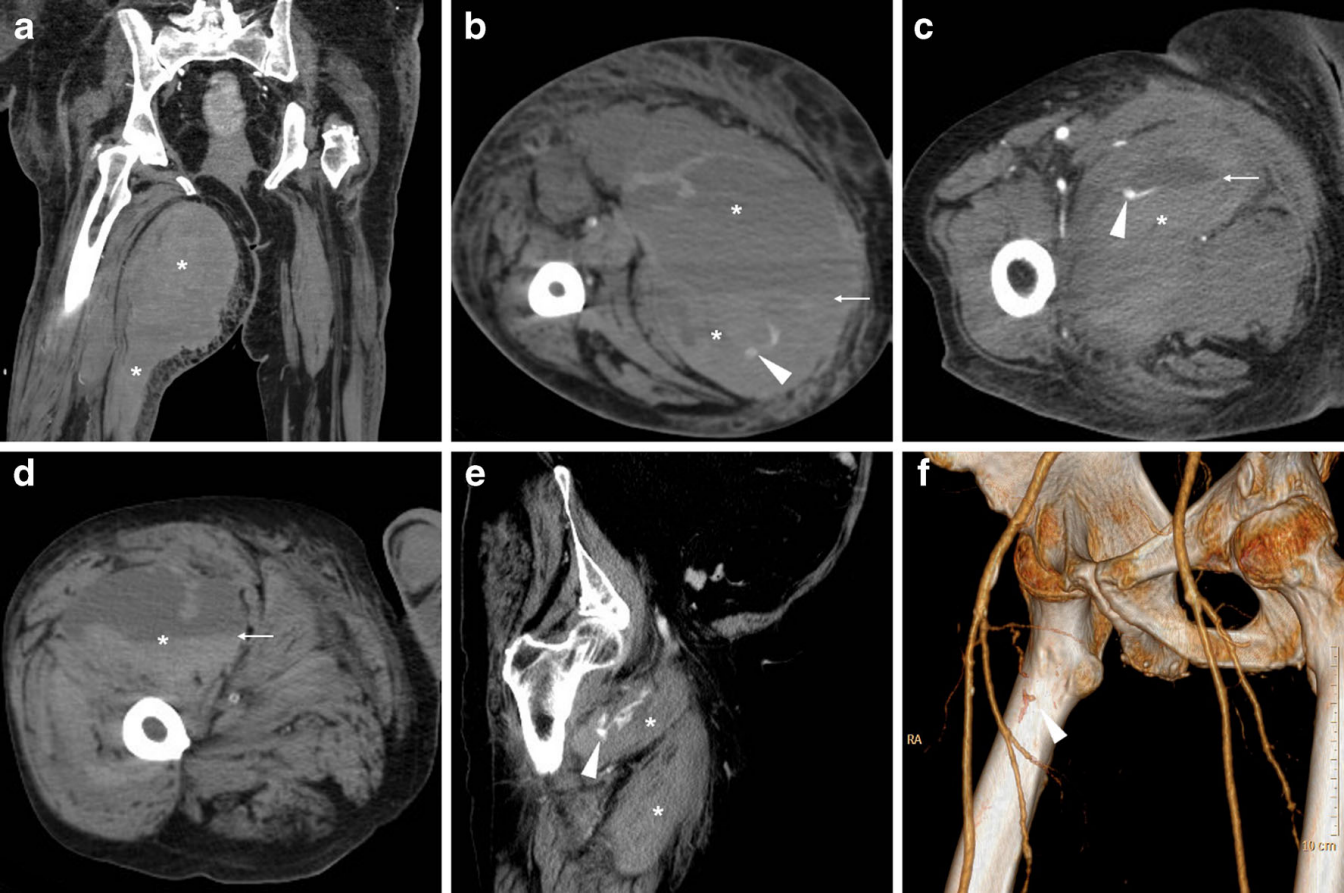
Three cases of thigh muscle haemorrhages. A-B) Large, actively bleeding haematoma (*) following percutaneous coronary intervention (PCI), occupying the entire medial muscular compartment of the right thigh, with mixed attenuation values and fluid-fluid level (thin arrow in B) and active CM extravasation (arrowhead in B). C) Sizeable (8 × 5 cm) adductor muscle haematoma of the right thigh after CA, with fluid-fluid level (thin arrow) and strongly hyperdense CM extravasation focus (arrowhead). D-F) Haematoma (*) of the quadriceps muscle with fluid-fluid level (thin arrow in D) five days after transcatheter ablation for atrial flutter, attributed to accidental arterial puncture as demonstrated by CM leakage (arrowheads) in coronal (E) and 3D volume-rendering (F) images

**Fig. 4:**
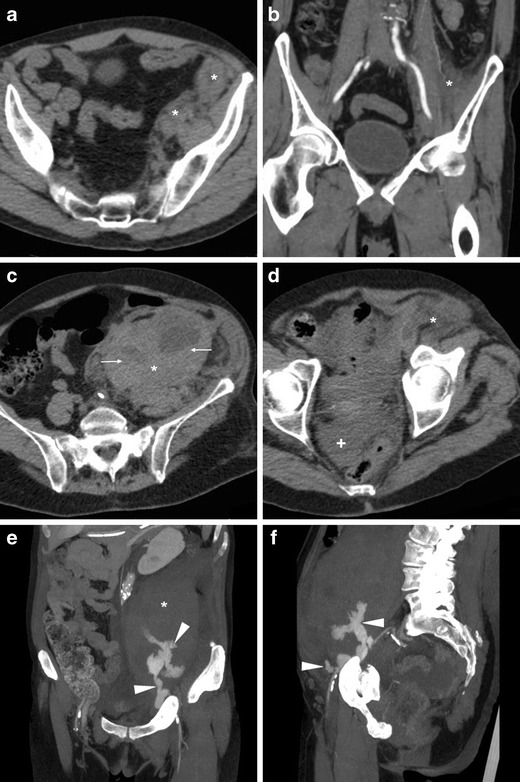
Intra-abdominal haemorrhages. A-B) In a 66-year-old man with severe peripheral artery disease (PA), shortly after percutaneous transluminal angioplasty (PTA) and stenting of the left femoral artery with ipsilateral vascular access, precontrast (A) CT images showed high-attenuation subperitoneal effusion (*) along the left iliac muscle, without active bleeding at CT-angiography (B), which was treated conservatively. C-F) In a 75-year-old woman with obliterating PAD, following PTA and stenting of the right superficial femoral artery via left CFA access, precontrast images (C, D) showed extensive, mass-like left retroperitoneal haematoma (*) with characteristic fluid-fluid levels (thin arrows), mild haemoperitoneum (+) in the pelvic cul-de-sac. CT angiography with maximum-intensity projection (MIP) images (E, F) showed extraluminal CM flowing from the site of arterial injury, indicative of active bleeding. Note displaced ipsilateral kidney. Emergency surgery including retroperitoneal decaillotage was required to seal the leaking femoral artery

From the injured CFA, post-catheterisation haemorrhage may either collect locally at the groin (Fig. 2), or extend downwards in the adductor or quadriceps thigh muscles (Fig. 3) or upwards in the pelvis (Fig. 4). The imaging diagnosis of haematoma relies on the identification of its characteristic precontrast hyperattenuation: hyperacute blood measures 40–60 Hounsfield Units (HU) due to its high protein content, which becomes even denser (60..80 HU) from clotting within a few hours and thus appears hyperdense compared to normal muscles. The involved muscles may be more or less enlarged compared to the contralateral ones. Subsequently, progressive haemoglobin lysis leads to a characteristic mixed-density appearance including geographic areas of lower attenuation and fluid-fluid levels (the so-called “haematocrit sign”) corresponding to different blood components stratified because of dependent settling of denser cellular elements [19].

If not contraindicated by impaired renal function or history of allergy, administration of iodinated contrast medium (CM) is warranted to detect ongoing bleeding, a finding which requires directed endovascular or surgical treatment. Arterial CT angiography is better acquired using high-flow (>3 ml/s) injection and automatic bolus-triggering technique with the region of interest placed in the infrarenal aorta and >100 HU cut-off. Active CM extravasation (Figs. 2, 3, 4) may be identified on either arterial or portal venous phase acquisitions depending on the injured vessel, and is best visualised using maximum intensity projection (MIP) or three-dimensional volume-rendering reconstructions [19].

### Pseudoaneurysm

Iatrogenic PA develop secondarily to failed sealing of an arterial puncture, from which blood dissects nearby causing the formation of a perfused “sac” contained by the media or adventitia, sometimes by soft tissues only. PA differs from haematoma because it is encapsulated and communicates with the arterial lumen [20, 21].

**Fig. 5:**
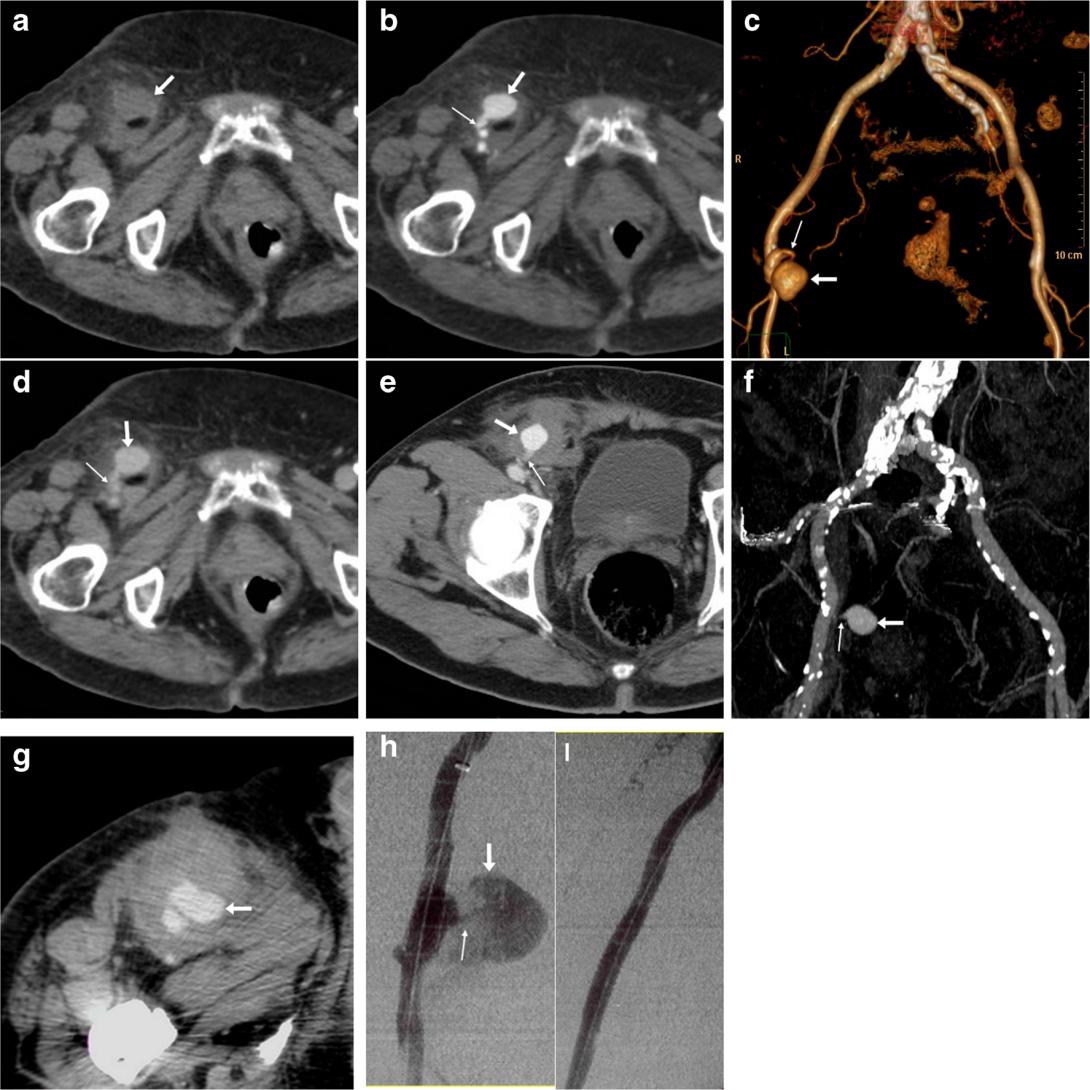
Three cases of right CFA pseudoaneurysms (PA). A-D) In an 87-year-old diabetic woman suffering from blood loss following PCI precontrast (A), CT angiography (B, volume-rendering C) and venous-phase (D) images showed small-sized (2 cm) round-shaped PA (arrows) and a short feeding tract (thin arrows) communicating with the CFA. E-F) In a 75-year-old man treated with thoracic endovascular aortic repair, CT-angiography (E) and MIP reconstruction (F) showed characteristic appearance of a PA (arrows) surrounded by blood, without active CM extravasation. Both occurrences were treated conservatively. G-I) In a 70-year-old man, CT angiography (G) showed a 5-cm right CFA PA (arrow), which was then confirmed angiographically (I) including optimal visualisation of thin connecting tract (thin arrows) and successfully treated with covered stent placement (I)

Sometimes clinically silent, PSA often manifest with tenderness and swelling at the site of arterial puncture. A pulsatile mass with associated palpable thrill or audible bruit is strongly suggestive for the diagnosis. Traditionally, the hallmark CDUS appearance is the “to-and-from” waveform through the feeding tract (FT). At CT angiography PA are demonstrated as round-shaped, peripherally haemorrhagic structures, internally perfused by CM from the CFA (Fig. 5). The length and calibre of the FT should be assessed, as it may impact the therapeutic choice [22, 23].

**Fig. 6:**
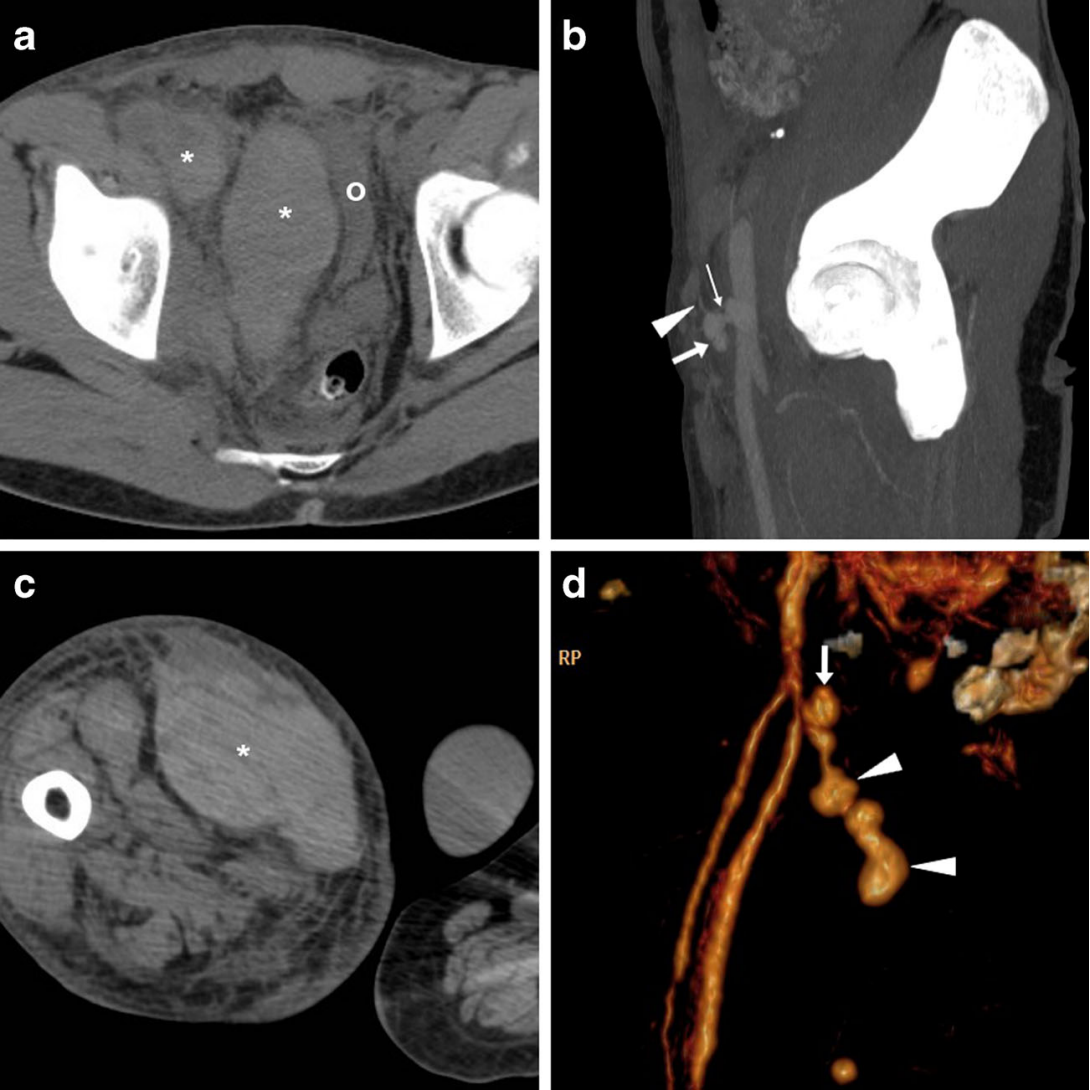
Two cases of right CFA PA complicated by bleeding. A-B) in a 29-year-old man on haemodialysis, suffering from pelvic pain four days after CA, precontrast (A) CT showed ample, high-attenuation retroperitoneal haematoma (*) compressing the urinary bladder (o). CT angiography MIP (B) showed PA (arrow) with thin feeding tract (thin arrow) and CM extravasation (arrowhead), which required immediate surgical treatment. C-D) In a 75-year-old man with myocardial ischaemia, 48 h after complex PCI CT detected a vast adductor muscle haematoma (*) caused by a small CFA PA (arrow volume-rendering image D) with CM extravasation (arrowheads), which required surgical repair

The key and most feared complication of a PA is rupture in either retroperitoneum or upper thigh, which depends on its size and is heralded by non-contained CM extravasation (Fig. 6). Other complications include pain from compression neuropathy, distal venous thrombosis, and limb ischaemia [5, 20, 21].

### Rare vascular complications

Other VASC following CFA access include AVF, arterial occlusion and dissection (Table 1). Their manifestations may be similar to those of a groin PA. Alternatively, these injuries may cause limited complaints and be incidentally discovered later [2–5, 16].

**Fig. 7:**
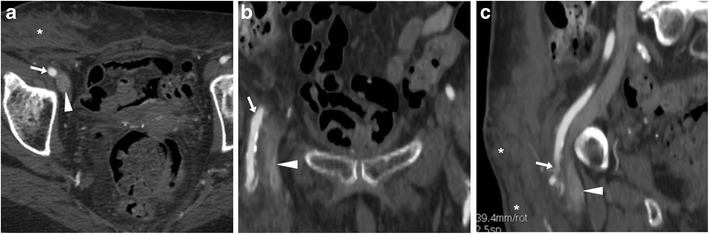
Arterio-venous fistula (AVF) observed 24 h after CA: multiplanar CT-angiographic images showed subcutaneous haematoma (*) and simultaneous opacification of the CFA (arrows) and adjacent femoral vein (arrowheads) consistent with AVF

**Fig. 8:**
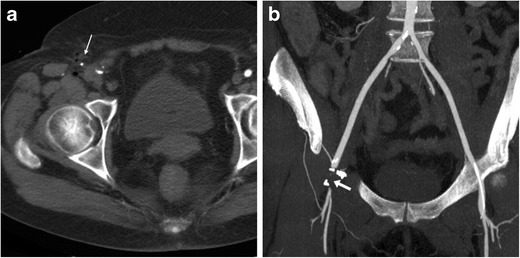
Segmental occlusion of the punctured right CFA in a 65-year-old woman suffering from acute lower limb ischaemia following transarterial mitral valve replacement. Despite inconclusive findings on precontrast CT images (thin arrow in a, note similarity with Fig.1), CT angiography (B) showed non-opacification of a 2-cm segment of the CFA (arrow). Surgical arteriotomy and endoarterectomy was immediately performed

Resulting from puncture-induced connection between the accessed CFA and the adjacent femoral vein, AVF manifests at CT angiography by synchronous luminal opacification (Fig. 7). Occlusion of the CFA is optimally demonstrated in its longitudinal extent as segmental non-opacification of the arterial lumen (Fig. 8). The rare dissection is heralded by the identification of an intimal flap separating the true from the false lumen in the CFA or iliac artery [16].

### Management

Traditionally, manual compression represented the “gold standard” in achieving haemostasis at an arterial access site. In the majority of VASC cases, further endovascular or surgical treatment is unnecessary, and a watchful conservative approach is sufficient. Most bleeding complications are self-limiting and are treated by re-application of pressure dressings. In major haemorrhage, haemodynamic stabilisation with blood transfusions is required. Endovascular therapies effectively use covered stents to seal active CFA bleeding, but may be limited by the considerable risk of bend-induced stent deformation at the level of the groin [3, 24].

According to the American College of Cardiology guidelines, in small-sized asymptomatic PA (up to 3 cm) and AVF a conservative approach is generally justified. PA with shorter (>1 cm) and wider FT close less easily compared to thin, long (>1 cm) ones. Therapeutic options include ultrasound-guided compression, percutaneous instillation of procoagulants (thrombin) into the aneurysmal sac, and stent implantation. Surgical revision is nowadays reserved for those cases refractory to conservative treatment. Finally, iatrogenic dissections may heal spontaneously or require endovascular treatment via contralateral or brachial access. Whereas self-expanding stents are used in the external iliac arteries, CFA dissection requires balloon angioplasty, since stents placed there are at high risk of fracture [5, 16, 20, 25, 26].

### Use of vascular closure devices (VCD)

During the last decade, VCD have been extensively investigated to improve haemostasis. Compared to manual compression, CD consistently achieved earlier mobilisation, and decreased patient discomfort and hospital stay. Although discussion of VCD types is beyond the scope of this article, they can be categorised as metal clips, suture-based, and collagen-plug devices. However, use of first-generation VCDs remains controversial due to a combination of factors, namely: a) the increased risk of local infections reported with AngioSeal and Perclose; b) the possibility of masking an ongoing bleeding; c) the possible development of lower limb ischaemia from device entrapment, dissection or thrombosis secondary to reaction to collagen plugs [11, 12] [13, 27, 28].

## Complications of iatrogenic and self-inflicted venous access

### Vascular complications after venous cannulation and interventions

**Fig. 9:**
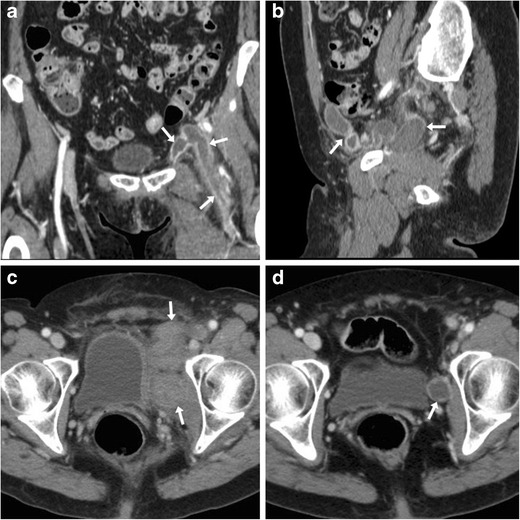
Endovenous heat-induced thrombosis (EHIT) in a 55-year-old woman experiencing left-sided groin pain, tenderness and swelling three days after endovenous laser ablation of the ipsilateral small saphenous vein. Multiplanar contrast-enhanced CT images (A, B) panoramically showed the full extent of iliac-femoral EHIT including the ipsilateral inferior hypogastric and obturator veins, with mild dilatation, luminal non-opacification and prominent enhancement of venous walls (arrows). One week later, repeated CT (C) showed persistent thrombosis of proximal femoral and obturator veins with disappeared mural enhancement (arrows) and increased mass effect on the urinary bladder. Follow-up CT three months later (D) showed recanalisation of the left femoral vein and persistence of chronic obturator vein thrombosis [Adapted from Open Access ref. no. [38]]

Following endovascular interventions performed via femoral venous access, haemorrhage (Fig. 3D-F) occurs rarely (0.3–1% of all procedures) compared to transarterial ones. Conversely, deep venous thrombosis (DVT) is relatively common (reported in up to 21% of patients) and often asymptomatic. In the electrophysiology setting, the incidence of DVT is lower (0.33% versus 2.38%) following treatment of atrial fibrillation (AF) compared to non-AF procedures, due to routine periprocedural anticoagulation. Albeit most instances are initially diagnosed using CDUS, using an appropriate CT-venography technique with delayed post-CM acquisition, multidetector CT effectively detects DVT in iliac and femoral veins with the usual appearance as luminal non-opacification and filling defects, causing variable degrees of venous enlargement [8]. A peculiar form of DVT is endovenous heat-induced thrombosis (EHIT), which represents the most feared complication (1% incidence within 72 h) of endovenous laser ablation (ELA), and requires anticoagulation to prevent development of pulmonary embolism. ELA is a minimally invasive treatment for lower-extremity venous insufficiency, which achieves venous obliteration by heat-induced irreversible damage to the vein lining, followed by fibrotic sealing of the lumen. CT allows for a comprehensive evaluation of EHIT proximal extension, which is categorised as grade 1 (thrombosis below the saphenous-femoral junction), 2, 3 and 4 respectively corresponding to thrombus occupying <50%, >50% or occluding the femoral vein diameter (Fig. 9) [29–31].

### Iatrogenic and self-inflicted groin infections

**Fig. 10:**
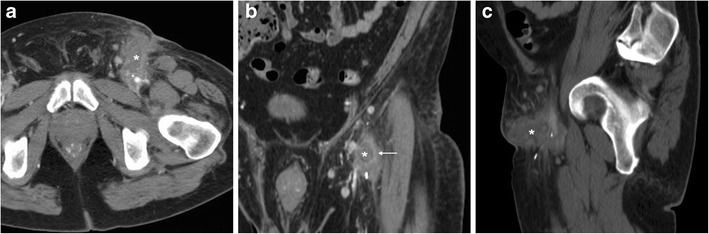
Groin abscess developing from superinfection of an iatrogenic haematoma in a 56-year-old man with acute coronary syndrome treated with PCI. Axial (A) and coronal (B) contrast-enhanced CT images showed hypoattenuating collection (*) with peripheral enhancement, extending ventrally from the CFA course. Antibiotics ultimately relieved the abscess

Compared to vascular injuries, iatrogenic groin infections have a similar incidence (below 1%) but tend to present later. Local cellulitis and abscesses may develop from superinfection of unrecognised or conservatively treated haematomas, and appear at CT as persistent inflammatory fat stranding and hypoattenuating collections with peripheral “ring” enhancement (Fig. 10) [16, 22, 23].

Furthermore, vascular and soft-tissue infections represent well-known complications of self-inflicted injection at the groin for recreational drug use. The risk is increased by non-sterile technique and by human immunodeficiency virus (HIV) infection [32]. Septic thrombophlebitis (STP) manifests with unspecific lower abdominal, flank or groin pain, leg oedema, and spiking fever despite broad-spectrum antibiotics, associated with variable leukocytosis and abnormal inflammatory markers. At haemocultures, *Staphylococcus aureus* is the most common causative organism [33, 34].

**Fig. 11:**
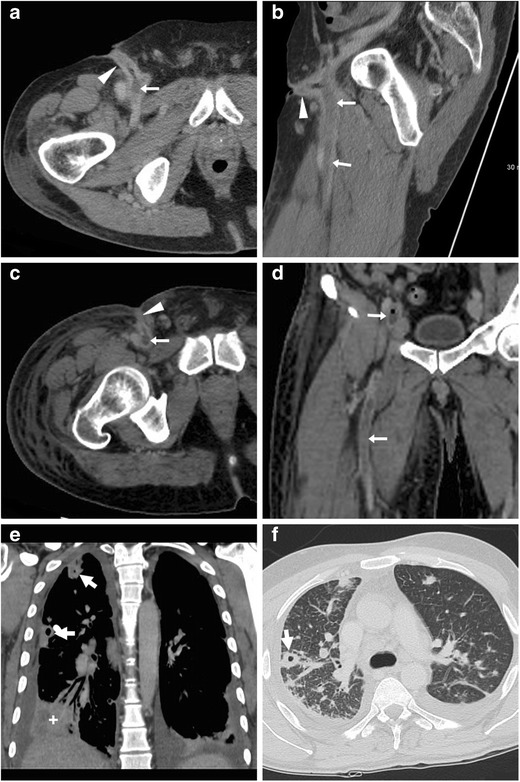
Two different cases of septic thrombophlebitis (STP) after intravenous drug injection. A-B) in a 57-year-old HIV-seropositive male patient, contrast-enhanced CT images showed strongly enhancing “tram-track” fistula (arrowheads) along the puncture track, and similar mural enhancement of the thrombosed ipsilateral femoral vein (arrows). Pus culture from draining orifice tested positive for Methycillin-sensitive *Staphylococcus aureus*. C-F) in a 34-year-old man with high fever, right thigh swelling, bloody and purulent drainage at the groin. The non-opacified right common, external iliac and femoral veins (arrows in C, D) showed prominent wall enhancement and some endoluminal gas bubbles. A “tram-track” fistula connected to the depressed injection site (arrowhead in C). Additionally, body CT showed pleural effusions, lung base consolidation (+), mediastinal adenopathies and subpleural lesions with central cavitations consistent with septic emboli (thick arrows in E, F). Blood and groin pus cultures revealed polymicrobial infection, which required two months of antibiotics and intensive care hospitalisation [Adapted with permission from ref. no. [39]]

Albeit chronic, non-occlusive thrombosis is commonly found in intravenous drug users at their usual injection site, STP is diagnosed on the basis of venous enlargement with partially or entirely non-opacified lumen, occasional intraluminal gas bubbles, thickened and/or strongly enhancing venous wall and inflammatory “stranding” of the surrounding fat planes (Fig. 11) [33, 35, 36]. If untreated, STP may be further complicated by septic embolisation to distant organs, most usually the lungs (Fig. 11E-F) [35].

### Sequelae of iatrogenic and self-inflicted venous injuries

**Fig. 12:**
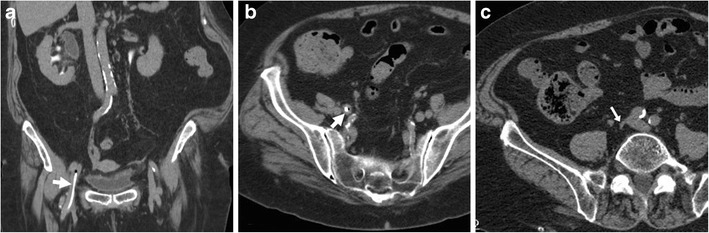
Long-term venous collapse following central venous catheterisation. On multidetector CT-urography (A, B) performed to investigate urosepsis in a 76-year-old woman, a right femoral CVC (thick arrows) is present. A year later, unenhanced CT image (C) incidentally showed very thin lumen of the ipsilateral common (arrow) and external iliac veins

**Fig. 13:**
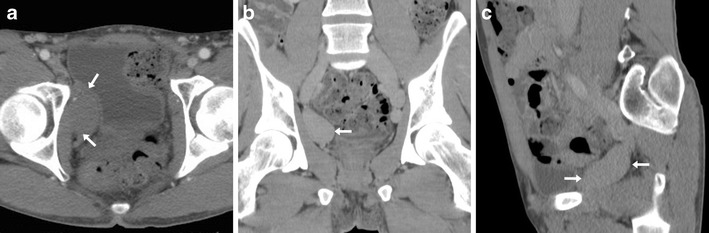
Post-thrombotic aneurysmal dilatation of the right hypogastric vein in a 34-year-old male intravenous drug user with long-standing history of lower limb thrombophlebitis. Requested to investigate sepsis and suspected deep venous thrombosis, CT showed a well-demarcated ovoid mass lesion abutting the right obturator muscle, measuring 30–35 HU precontrast attenuation (A). The lesion compressed the urinary bladder, enhanced homogeneously and synchronously with venous structures (B, C). Surgical repair was unnecessary considering absence of complications [Adapted from Open Access ref. no. [40]]

Long-term consequences of venous thrombosis include collapsed veins (Fig. 12) and the very rare venous aneurysms (VA). Multidetector CT is required to correctly characterise VA as segmental dilatation of venous structures, with enhancement paralleling that of other veins in the same system (Fig. 13), thus avoiding misinterpretation as lymphadenopathy, retroperitoneal or adnexal tumours which may result in dangerous procedures such as biopsy [37].

## Conclusion

Despite the shift towards establishing vascular access via the radial artery instead of the CFA, cannulation of the latter remains frequent in coronary and non-cardiac interventions. In recent years, we have witnessed a significant increase in urgent requests for pelvic and femoral CT angiography studies in patients with suspected iatrogenic injuries. Despite the disadvantages of ionising radiation and need for intravenous contrast, multidetector CT provides rapid and effective triage of troublesome groin complications secondarily to femoral arterial or venous access, which is required for correct choice between conservative, endovascular and surgical treatment. Familiarity with CT findings of iatrogenic and self-inflicted VASC and infections at the groin is warranted.

## References

[1] Means G, End C, Kaul P (2017). Management of percutaneous coronary intervention complications. Curr Treat Options Cardiovasc Med.

[2] Dencker D, Pedersen F, Engstrom T (2016). Major femoral vascular access complications after coronary diagnostic and interventional procedures: a Danish register study. Int J Cardiol.

[3] Dencker D, Taudorf M, Luk NH (2016). Frequency and effect of access-related vascular injury and subsequent vascular intervention after transcatheter aortic valve replacement. Am J Cardiol.

[4] Tonnessen BH (2011). Iatrogenic injury from vascular access and endovascular procedures. Perspect Vasc Surg Endovasc Ther.

[5] Mlekusch W, Mlekusch I, Sabeti-Sandor S (2016). Vascular puncture site complications - diagnosis, therapy, and prognosis. Vasa.

[6] Kalish J, Eslami M, Gillespie D (2015). Routine use of ultrasound guidance in femoral arterial access for peripheral vascular intervention decreases groin hematoma rates. J Vasc Surg.

[7] Akaraborworn O (2017). A review in emergency central venous catheterization. Chin J Traumatol.

[8] Burstein B, Barbosa RS, Kalfon E (2017). Venous thrombosis after electrophysiology procedures: a systematic review. Chest.

[9] Ge X, Cavallazzi R, Li C, et al (2012) Central venous access sites for the prevention of venous thrombosis, stenosis and infection. Cochrane Database Syst Rev: Cd004084 10.1002/14651858.CD004084.pub3 PMC6516884 22419292

[10] McGee DC, Gould MK (2003). Preventing complications of central venous catheterization. N Engl J Med.

[11] Franco J, Motaganahalli R, Habeeb M (2009). Risk factors for infectious complications with angio-seal percutaneous vascular closure devices. Vascular.

[12] Smith TP, Cruz CP, Moursi MM (2001). Infectious complications resulting from use of hemostatic puncture closure devices. Am J Surg.

[13] Toursarkissian B, Mejia A, Smilanich RP (2001). Changing patterns of access site complications with the use of percutaneous closure devices. Vasc Surg.

[14] Hope VD, Scott J, Cullen KJ (2015). Going into the groin: injection into the femoral vein among people who inject drugs in three urban areas of England. Drug Alcohol Depend.

[15] Mackenzie AR, Laing RB, Douglas JG (2000). High prevalence of iliofemoral venous thrombosis with severe groin infection among injecting drug users in North East Scotland: successful use of low molecular weight heparin with antibiotics. Postgrad Med J.

[16] Kolluri R, Fowler B, Nandish S (2013). Vascular access complications: diagnosis and management. Curr Treat Options Cardiovasc Med.

[17] Sobolev M, Slovut DP, Lee Chang A (2015). Ultrasound-guided catheterization of the femoral artery: a systematic review and meta-analysis of randomized controlled trials. J Invasive Cardiol.

[18] Smilowitz NR, Kirtane AJ, Guiry M (2012). Practices and complications of vascular closure devices and manual compression in patients undergoing elective transfemoral coronary procedures. Am J Cardiol.

[19] Tonolini M, Ippolito S, Patella F (2012). Hemorrhagic complications of anticoagulant therapy: role of multidetector computed tomography and spectrum of imaging findings from head to toe. Curr Probl Diagn Radiol.

[20] Stone PA, Campbell JE, AbuRahma AF (2014). Femoral pseudoaneurysms after percutaneous access. J Vasc Surg.

[21] Tisi PV, Callam MJ (2013) Treatment for femoral pseudoaneurysms. Cochrane Database Syst Rev: Cd004981 10.1002/14651858.CD004981.pub4 PMC12066186 24293322

[22] Shadbolt CL, Heinze SB, Dietrich RB (2001) Imaging of groin masses: inguinal anatomy and pathologic conditions revisited. Radiographics 21 Spec No:S261–271 10.1148/radiographics.21.suppl_1.g01oc17s261 11598262

[23] Bhosale PR, Patnana M, Viswanathan C (2008). The inguinal canal: anatomy and imaging features of common and uncommon masses. Radiographics.

[24] Arat A, Turkbey B, Cil BE (2007). Emergent treatment of an iatrogenic arterial injury at femoral puncture site with Symbiot self-expanding PTFE-covered coronary stent-graft. Australas Radiol.

[25] Kontopodis N, Tsetis D, Tavlas E (2016). Ultrasound guided compression versus ultrasound guided thrombin injection for the treatment of post-catheterization femoral pseudoaneurysms: systematic review and meta-analysis of comparative studies. Eur J Vasc Endovasc Surg.

[26] Tsetis D (2010). Endovascular treatment of complications of femoral arterial access. Cardiovasc Intervent Radiol.

[27] Robertson L, Andras A, Colgan F (2016). Vascular closure devices for femoral arterial puncture site haemostasis. Cochrane Database Syst Rev.

[28] Schwartz BG, Burstein S, Economides C (2010). Review of vascular closure devices. J Invasive Cardiol.

[29] Malgor RD, Gasparis AP, Labropoulos N (2016). Morbidity and mortality after thermal venous ablations. Int Angiol.

[30] Memetoglu ME, Kurtcan S, Erbasan O (2012). Endovenous ablation with a 940 nm laser for the treatment of great saphenous vein insufficiency: short- to mid-term results. Diagn Interv Radiol.

[31] Dermody M, Schul MW, O'Donnell TF (2014) Thromboembolic complications of endovenous thermal ablation and foam sclerotherapy in the treatment of great saphenous vein insufficiency. Phlebology 10.1177/0268355514529948 24699720

[32] Restrepo CS, Diethelm L, Lemos JA (2006). Cardiovascular complications of human immunodeficiency virus infection. Radiographics.

[33] Garcia J, Aboujaoude R, Apuzzio J (2006). Septic pelvic thrombophlebitis: diagnosis and management. Infect Dis Obstet Gynecol.

[34] Chirinos JA, Garcia J, Alcaide ML (2006). Septic thrombophlebitis: diagnosis and management. Am J Cardiovasc Drugs.

[35] Hagan IG, Burney K (2007). Radiology of recreational drug abuse. Radiographics.

[36] Huang JS, Ho AS, Ahmed A (2011). Borne identity: CT imaging of vascular infections. Emerg Radiol.

[37] Calligaro KD, Ahmad S, Dandora R (1995). Venous aneurysms: surgical indications and review of the literature. Surgery.

[38] Tonolini M (2017) Endovenous heat-induced thrombosis following saphenous laser ablation {Online}. EuroRAD Case 15150

[39] Tonolini M (2012) Iliaco-femoral septic thrombophlebitis from intravenous drug use: MDCT diagnosis and follow-up {Online}. EuroRAD Case 10288

[40] Tonolini M (2016) Post-thrombotic aneurysmal dilatation of the hypogastric vein {Online}. EuroRAD Case 13432

